# Rationale for the Potential Use of Recombinant Activated Factor VII in Severe Post-Partum Hemorrhage

**DOI:** 10.3390/jcm13102928

**Published:** 2024-05-16

**Authors:** Nándor Ács, Wolfgang C. Korte, Christian C. von Heymann, Jerzy Windyga, Jan Blatný

**Affiliations:** 1 Department of Obstetrics and Gynaecology, Semmelweis University, H-1082 Budapest, Hungary; 2 Centre for Laboratory Medicine, Haemostasis and Haemophilia Centre, CH-9001 St. Gallen, Switzerland; 3 Department of Anaesthesia, Intensive Care Medicine, Emergency Medicine and Pain Therapy, Vivantes Klinikum in Friedrichshain, DE-10249 Berlin, Germany; 4 Department of Haemostasis Disorders and Internal Medicine, Laboratory of Haemostasis and Metabolic Diseases, Institute of Haematology and Transfusion Medicine, 02-776 Warsaw, Poland; 5 Department of Paediatric Oncology, University Hospital Brno, and Masaryk University, 613 00 Brno, Czech Republic

**Keywords:** massive post-partum hemorrhage, Novo Seven, obstetric hemorrhage, rFVIIa mode of action

## Abstract

Severe post-partum hemorrhage (PPH) is a major cause of maternal mortality worldwide. Recombinant activated factor VII (rFVIIa) has recently been approved by the European Medicines Agency for the treatment of severe PPH if uterotonics fail to achieve hemostasis. Although large randomized controlled trials are lacking, accumulated evidence from smaller studies and international registries supports the efficacy of rFVIIa alongside extended standard treatment to control severe PPH. Because rFVIIa neither substitutes the activity of a missing coagulation factor nor bypasses a coagulation defect in this population, it is not immediately evident how it exerts its beneficial effect. Here, we discuss possible mechanistic explanations for the efficacy of rFVIIa and the published evidence in patients with severe PPH. Recombinant FVIIa may not primarily increase systemic thrombin generation, but may promote local thrombin generation through binding to activated platelets at the site of vascular wall injury. This explanation may also address safety concerns that have been raised over the administration of a procoagulant molecule in a background of increased thromboembolic risk due to both pregnancy-related hemostatic changes and the hemorrhagic state. However, the available safety data for this and other indications are reassuring and the rates of thromboembolic events do not appear to be increased in women with severe PPH treated with rFVIIa. We recommend that the administration of rFVIIa be considered before dilutional coagulopathy develops and used to support the current standard treatment in certain patients with severe PPH.

## 1. Severe Post-Partum Hemorrhage

Severe post-partum hemorrhage (PPH) is the one of the leading causes of maternal morbidity and mortality, and blood loss of 1500 mL or more occurs in approximately 3% of all births worldwide [1]. Currently, the most frequent causative factor for severe PPH is believed to be uterine atony (70–80% of cases) [2,3]. Other causes include placental retention (approx. 10–30%), uterine trauma and laceration of the cervix or vagina (15–20%), and less frequently, coagulopathy (due to utilization, breakdown or dilution of coagulation factors, ~1%) or undiagnosed or inadequately managed inherited or acquired bleeding disorders (e.g., von Willebrand disease or acquired hemophilia A) [3].

**Figure 1:**
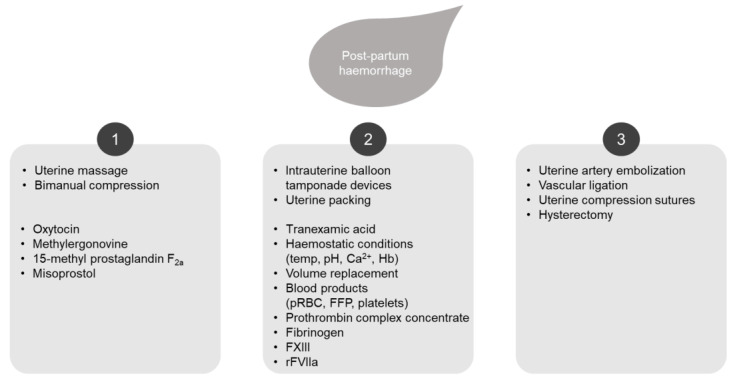
Management of severe post-partum hemorrhage using a multistep approach, including the potential role of rFVIIa. First-line treatment options include mechanical and acute medical management with uterotonics (1). If uterotonics fail to control the bleeding, second-line options include volume replacement, hemostatic agents, blood products and non-invasive surgical procedures (2). If bleeding persists, invasive procedures may be required (3). FFP, fresh frozen plasma; FXIII, factor XIII; Hb; hemoglobin; pRBC, packed red blood cells; rFVIIa, recombinant activated factor VII.

PPH is commonly managed using a multistep approach that combines pharmacological and mechanical measures (Figure 1) [4,5]. First-line treatment options include uterotonics. If uterotonics fail to control the bleeding, volume replacement therapy and various hemostatic agents, blood products and non-invasive surgical procedures such as balloon or hemostatic dressing tamponades are applied as second-line treatment. Additional treatment is needed if bleeding persists in order to prevent hemorrhagic shock. This includes invasive procedures such as uterine compression sutures or arterial ligation, arterial embolization and, ultimately, hysterectomy as a last resort [4].

A number of evidence-based international and national treatment guidelines for PPH exist, which should be implemented in emergency situations to guide treatment decisions [1,4,6,7]. While some treatments are not readily available at all medical facilities, non-invasive treatment options may facilitate transport to larger centers with appropriate radiological and surgical facilities and blood banks or, in the best case, prevent the need for further treatment. This expert opinion aims to build on existing guidelines by outlining a rationale for the timing of the administration of recombinant activated factor VII (rFVIIa) during the treatment of certain cases of severe PPH.

Recombinant FVIIa has been used off-label in severe PPH based on empirical reports for over 20 years [8]. In Europe, rFVIIa (NovoSeven^®^, Novo Nordisk, Bagsværd, Denmark) was approved in 2022 for use in patients with severe PPH if uterotonics fail to achieve hemostasis [9]. In addition to severe PPH, rFVIIa is approved for the treatment of patients with congenital hemophilia A or B with inhibitory alloantibodies against coagulation factor (F) VIII/FIX, acquired hemophilia, congenital FVII deficiency and in patients with Glanzmann’s thrombasthenia for the treatment of bleeding episodes and the prevention of bleeding during surgical interventions or surgical procedures [10].

## 2. rFVIIa Mechanism of Action

**Figure 2:**
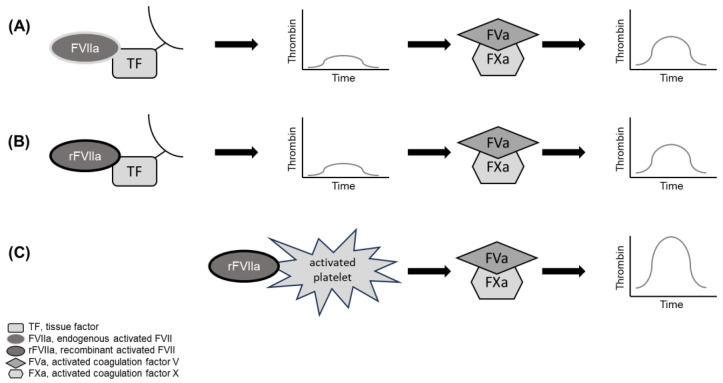
Hypothesized rFVIIa mechanism of action. Endogenous FVIIa forms a complex with tissue factor to initiate hemostasis (**A**). Recombinant FVIIa administered at pharmacological doses is over 200 times the level of endogenous FVIIa and binds to tissue factor (**B**) and with low affinity to activated platelets (**C**). On the activated platelet surface, rFVIIa induces a TF-independent increase in thrombin generation, bypassing the intrinsic coagulation pathway. In parallel, a TF-dependent pathway also promotes thrombin generation. FVa, activated factor V; FVIIa, activated factor VII; FXa, activated factor X; rFVIIa, recombinant activated factor VII; TF, tissue factor.

The rFVIIa mechanism of action is well understood (Figure 2). Endogenous FVIIa forms a complex with tissue factor (TF) to initiate hemostasis [11]. Recombinant FVIIa is administered at pharmacological doses, which are over 200 times higher than endogenous FVIIa levels and thus binds with low affinity to activated platelets as well as to TF and FX, thereby activating and releasing FXa [12]. On the activated platelet surface, rFVIIa then induces a partially TF-independent increase in thrombin generation, bypassing the intrinsic coagulation pathway that requires FVIII and FIX. Prothrombin is converted into thrombin and soluble fibrin is generated, which is cross-linked by the transglutaminase FXIIIa to form a stable fibrin clot. The exposure of TF to the vasculature and the activation of platelets occurs at the site of injury. This explains why rFVIIa exerts only part of its effect through the systemic activation of coagulation [12].

## 3. rFVIIa Use in Different Pathophysiological Conditions

Recombinant FVIIa has been used off-label to treat several pathophysiological conditions other than severe PPH. Here, we review findings and conclusions drawn from these studies and discuss features specific to PPH pathophysiology.

Following severe trauma, patients may develop trauma-induced coagulopathy [13]. This condition may encompass several factors, including tissue trauma, shock, the activation of hyperfibrinolysis, hemodilution, hypothermia, acidosis and inflammation [14]. Some of these factors may be encountered in severe PPH as well, including tissue trauma (though less pronounced than in trauma patients), hypoxia, shock and hemodilution.

The effectiveness of rFVIIa in reducing blood loss has been reported in patients following traumatic injury [15]. Laboratory tests showed that thrombin generation is increased after the administration of rFVIIa in trauma patients [16]. Similarly, the administration of rFVIIa led to an increase in thrombin generation in patients undergoing prostatectomy in a small randomized controlled trial (RCT) [17]. While trauma-induced coagulopathy and severe PPH share some characteristics, these two scenarios are not fully comparable and are separated by important distinctions.

The pathophysiology of PPH is influenced by specific pregnancy-related features that include increased levels of many (e.g., FXIII, FVIII, Von Willebrand factor), albeit not all, coagulation factors [18]. This hypercoagulable state probably serves to protect the parturient from extensive blood loss during delivery, for example, due to uterine atony [19]. Severe PPH itself, volume replacement for hemodynamic stabilization and red blood cell transfusions aggravate the depletion and dilution of coagulation factors and platelets. Increased fibrinolytic activity, although not the main reason for low fibrinogen, has also been noted shortly after delivery, especially in patients with placental disruption [18]. Reduced levels of various other coagulation factors and platelets may lower but not abolish rFVIIa effectiveness. We hypothesize that by bypassing the classic coagulation pathway, rFVIIa acts locally on the surface of activated platelets and accelerates the local thrombin burst; however, additional studies in the setting of PPH are needed to further elucidate the specific mechanisms of rFVIIa activity in this population.

## 4. Efficacy of rFVIIa

The use of rFVIIa in approved, longstanding indications (hemophilia A or B with inhibitors, acquired hemophilia, congenital FVII deficiency and Glanzmann’s thrombasthenia) is highly efficacious, as has been shown in several RCTs [10,20]. The efficacy rate of rFVIIa in the treatment of severe bleeding and major surgery in patients with congenital hemophilia A and B with inhibitors is very high at around 90% [20].

Evidence from a number of small case series describing off-label use in severe PPH attested to the potential efficacy of rFVIIa in this setting. Overall, rFVIIa was able to stop or reduce bleeding in 85% of the reported patients, as was shown in a literature review by Franchini et al. [21]. The Australian and New Zealand Haemostasis Registry (ANZHR) collected data on 177 patients with severe PPH between 2000 and 2009 and reported that bleeding stopped or decreased in 64% of cases after the final dose of rFVIIa (76% of patients had received a single dose) [22]. In the Northern European Registry, improvements upon treatment with rFVIIa were recorded in 80% of women with PPH (n = 92) [23]. Similarly, Ahonen et al. reported a good response to treatment of severe PPH with rFVIIa in 17 and a moderate response in 3 out of 26 women (77% taken together) [24]. One RCT investigated rFVIIa use in patients with severe PPH (n = 84 women) after two uterotonics failed to achieve hemostasis and showed a reduction in the need for further therapeutic measures [25]. However, data from large, controlled studies are lacking.

In 2022, the European Medicines Agency (EMA) extended the labelled approval for rFVIIa to severe PPH when uterotonics are insufficient to achieve hemostasis. In the accompanying European Public Assessment Report (EPAR), data from the aforementioned RCT and four observational studies (OS) were re-analyzed with regards to efficacy and safety of rFVIIa use in severe PPH [26]. The rate of invasive procedures (arterial ligations, compression sutures, embolizations or hysterectomies), the primary endpoint chosen for analysis of the RCT data, was reduced by a relative 44.7% in the group of women exposed to rFVIIa compared with non-exposed women (n = 42 in each group, *p* < 0.0001), whereby this effect was driven primarily by a reduction in arterial ligations. This effect was not confirmed in OS-1 with propensity-score-matched control populations, while the results of OS-2 were inconclusive. OS-3 and OS-4 included rFVIIa-exposed women only and therefore did not have a comparative efficacy readout. Potential reasons for the different outcomes may include the small number of patients assessed and diverse study designs, patient populations and settings. It is also conceivable that the matching did not remove all confounding factors.

## 5. Safety of rFVIIa

Overall, rFVIIa is considered to have a good safety profile in the long-standing indications; even after 20 years on the market, the occurrence of thromboembolic events (TEs) is generally very rare [10]. Systematic reviews or rFVIIa trials outside hemophilia reported an increased risk of arterial TEs, specifically in patients over 65 years [27,28].

Parturient women per se have an increased risk of developing a TE until 3–6 weeks after delivery [29]. The risk of developing a venous TE during pregnancy ranges between an incidence of 0.5–2 per 1000 pregnancies [30]. In women with PPH, the risk for TEs is further increased [29]. It can therefore be challenging to establish causality between rFVIIa administration and a subsequent TE. Irrespective of this, anticoagulant strategies (or thromboprophylaxis) are recommended as the standard of care for women with PPH once the bleeding has stopped [1], regardless of clinical management strategy.

A meta-analysis of the safety of rFVIIa presented in the rFVIIa extension of indication EPAR showed that, in the overall population (RCT and four OS), the proportion of women with severe PPH and a TE was similar in rFVIIa-exposed and non-exposed women (1.5% versus 1.6%) [26]. The rate of TEs in rFVIIa-exposed women ranged from 0 to 4.3% across the individual studies included in the analysis. The report concluded that rFVIIa does not add any further risk of developing a TE in the setting of severe PPH, although not all the data included in the analysis were from RCTs.

For comparison, the incidence of TEs in three RCTs on fibrinogen substitution in PPH and severe PPH ranged from 0 to 3.6% [31,32,33]. In a large RCT on the use of tranexamic acid to prevent PPH (WOMAN), there was no difference observed in the rate of TEs (0.2 vs. 0.3% in the tranexamic acid and placebo groups, respectively) [34]. The sequential administration/combination of tranexamic acid and rFVIIa may be considered in order to enhance efficacy. A combination of both agents has been reported to be effective and show a favorable safety profile in the treatment of patients with hemophilia A with inhibitors, factor XI-deficiency and PPH [35,36,37]. Nonetheless, all patients who experience PPH should be carefully monitored for signs of potential TEs. Monitoring is also essential in patients who receive rFVIIa, as controlled and sufficiently powered data on the safety of rFVIIa in the setting of severe PPH are lacking.

## 6. Timing of rFVIIa Administration

Hemostatic conditions may deteriorate in the later course of severe PPH, leading to dilutional or consumptive coagulopathy. The updated label indicates rFVIIa treatment for severe PPH after the failure of uterotonics to achieve hemostasis, positioning rFVIIa earlier in the treatment algorithm rather than as a “last resort” treatment. It is conceivable that rFVIIa works best in those settings in which it is administered before dilutional and consumptive coagulopathy has fully developed. Overall, fibrinogen and platelets were above the recommended minimum levels in the RCT that demonstrated reduced rates of invasive procedures in women treated with rFVIIa [25]. Similarly, results from the international WOMAN trial suggested that the early (within 3 h) treatment of women with PPH with tranexamic acid is most effective [38]. Consensus statements from an expert panel include different clinical scenarios where rFVIIa may be considered as second-line treatment for severe PPH [39]. Additional clinical trials are required to confirm the ideal timing of rFVIIa administration in women with severe PPH.

## 7. Considerations for the Use of rFVIIa in Severe PPH

There are a number of considerations that may provide a rationale for the use of rFVIIa in certain patients with severe PPH. By bypassing the intrinsic coagulation pathway locally, rFVIIa may accelerate the natural thrombin burst in a tissue-factor-independent manner. The use of rFVIIa can help to reduce blood loss, transfusion requirements and invasive procedures, thereby preserving fertility and reducing the risk of post-operative complications. rFVIIa can be administered as a temporizing treatment, allowing transfer to an appropriate medical facility. In a resource-poor setting or in patients in whom blood products are not an option, rFVIIa may help to avoid extensive blood product and/or coagulation factor concentrate use [22]. To date, cost–benefit data for the use of rFVIIa in the context of severe PPH are unavailable [39] and this should be addressed in further studies on rFVIIa and severe PPH. No antagonistic or unwanted additive drug–drug interactions in clinical settings between rFVIIa and other approved medications/procedures used in severe PPH treatment have been reported [10,25,39].

We recommend that the administration of rFVIIa be considered in certain patients with severe PPH before dilutional coagulopathy develops who have failed to respond to uterotonics alongside other measures to stop hemorrhage. Based on a number of overall positive reports and approval by the EMA for rFVIIa use in severe PPH, administration may no longer be considered only as a last resort, including in settings without access to larger blood bank resources, coagulation factor concentrates and angiographic and surgical facilities [22]. Given the level of evidence available to date, further data on the efficacy and safety of rFVIIa in the management of severe PPH should be assessed in future studies and guidelines.

## 8. Conclusions

Overall, the efficacy profile of rFVIIa reported to date in severe PPH supports its administration in certain patients in whom initial standard treatment is insufficient. The earlier administration of rFVIIa in patients with severe PPH may be more effective than later administration in a more advanced coagulopathic state. The assumed mode of action and the clinical data gathered to date suggest a clinically acceptable safety profile for rFVIIa.

## Data Availability

Not applicable.

## References

[1] Annecke T., Lier H., Girard T., Korte W., Pfanner G., Schlembach D., Tiebel O., von Heymann C. (2022). Peripartum hemorrhage, diagnostics and treatment: Update of the S2k guidelines AWMF 015/063 from August 2022. Anaesthesiologie.

[2] Bateman B.T., Berman M.F., Riley L.E., Leffert L.R. (2010). The epidemiology of postpartum hemorrhage in a large, nationwide sample of deliveries. Anesth. Analg..

[3] Sentilhes L., Merlot B., Madar H., Sztark F., Brun S., Deneux-Tharaux C. (2016). Postpartum haemorrhage: Prevention and treatment. Expert Rev. Hematol..

[4] Committee on Practice Bulletins-Obstetrics (2017). Practice bulletin no. 183: Postpartum hemorrhage. Obstet. Gynecol..

[5] Kellie F.J., Wandabwa J.N., Mousa H.A., Weeks A.D. (2020). Mechanical and surgical interventions for treating primary postpartum haemorrhage. Cochrane Database Syst. Rev..

[6] Robinson D., Basso M., Chan C., Duckitt K., Lett R. (2022). Guideline No. 431: Postpartum hemorrhage and hemorrhagic shock. J. Obstet. Gynaecol. Can..

[7] Escobar M.F., Nassar A.H., Theron G., Barnea E.R., Nicholson W., Ramasauskaite D., Lloyd I., Chandraharan E., Miller S., Burke T. (2022). FIGO recommendations on the management of postpartum hemorrhage 2022. Int. J. Gynaecol. Obstet..

[8] Welsh A., McLintock C., Gatt S., Somerset D., Popham P., Ogle R. (2008). Guidelines for the use of recombinant activated factor VII in massive obstetric haemorrhage. Aust. N. Z. J. Obstet. Gynaecol..

[9] European Medicines Agency NovoSeven. https://www.ema.europa.eu/en/medicines/human/EPAR/novoseven.

[10] Neufeld E.J., Négrier C., Benchikh El Fegoun S., Cooper D.L., Rojas-Rios A., Seremetis S. (2018). Recombinant activated factor VII in approved indications: Update on safety. Haemophilia.

[11] Mann K.G., Krudysz-Amblo J., Butenas S. (2012). Tissue factor controversies. Thromb. Res..

[12] Hoffman M., Monroe D.M., Roberts H.R. (1998). Activated factor VII activates factors IX and X on the surface of activated platelets: Thoughts on the mechanism of action of high-dose activated factor VII. Blood Coagul. Fibrinolysis.

[13] Caspers M., Maegele M., Fröhlich M. (2018). Current strategies for hemostatic control in acute trauma hemorrhage and trauma-induced coagulopathy. Expert Rev. Hematol..

[14] Hess J.R., Brohi K., Dutton R.P., Hauser C.J., Holcomb J.B., Kluger Y., Mackway-Jones K., Parr M.J., Rizoli S.B., Yukioka T. (2008). The coagulopathy of trauma: A review of mechanisms. J. Trauma.

[15] Dutton R.P., Conti B.M. (2009). The role of recombinant-activated factor VII in bleeding trauma patients. Curr. Opin. Anaesthesiol..

[16] Yao D., Li Y., Wang J., Yu W., Li N., Li J. (2014). Effects of recombinant activated factor VIIa on abdominal trauma patients. Blood Coagul. Fibrinolysis.

[17] Friederich P.W., Henny C.P., Messelink E.J., Geerdink M.G., Keller T., Kurth K.H., Büller H.R., Levi M. (2003). Effect of recombinant activated factor VII on perioperative blood loss in patients undergoing retropubic prostatectomy: A double-blind placebo-controlled randomised trial. Lancet.

[18] McNamara H., Mallaiah S. (2019). Managing coagulopathy following PPH. Best Pract. Res. Clin. Obstet. Gynaecol..

[19] Vermeulen T., Van de Velde M. (2022). The role of fibrinogen in postpartum hemorrhage. Best Pract. Res. Clin. Anaesthesiol..

[20] Hedner U., Lee C.A. (2011). First 20 years with recombinant FVIIa (NovoSeven). Haemophilia.

[21] Franchini M., Franchi M., Bergamini V., Montagnana M., Salvagno G.L., Targher G., Lippi G. (2010). The use of recombinant activated FVII in postpartum hemorrhage. Clin. Obstet. Gynecol..

[22] Zatta A., McQuilten Z., Kandane-Rathnayake R., Isbister J., Dunkley S., McNeil J., Cameron P., Phillips L. (2015). The Australian and New Zealand Haemostasis Registry: Ten years of data on off-licence use of recombinant activated factor VII. Blood Transfus..

[23] Alfirevic Z., Elbourne D., Pavord S., Bolte A., Van Geijn H., Mercier F., Ahonen J., Bremme K., Bødker B., Magnúsdóttir E.M. (2007). Use of recombinant activated factor VII in primary postpartum hemorrhage: The Northern European registry 2000-2004. Obstet. Gynecol..

[24] Ahonen J., Jokela R., Korttila K. (2007). An open non-randomized study of recombinant activated factor VII in major postpartum haemorrhage. Acta Anaesthesiol. Scand..

[25] Lavigne-Lissalde G., Aya A.G., Mercier F.J., Roger-Christoph S., Chauleur C., Morau E., Ducloy-Bouthors A.S., Mignon A., Raucoules M., Bongain A. (2015). Recombinant human FVIIa for reducing the need for invasive second-line therapies in severe refractory postpartum hemorrhage: A multicenter, randomized, open controlled trial. J. Thromb. Haemost..

[26] Caram-Deelder C., McKinnon Edwards H., Zdanowicz J.A., van den Akker T., Birkegård C., Blatný J., van der Bom J.G., Colucci G., van Duuren D., van Geloven N. (2024). Efficacy and safety analyses of recombinant factor VIIa in severe post-partum hemorrhage. J. Clin. Med..

[27] Simpson E., Lin Y., Stanworth S., Birchall J., Doree C., Hyde C. (2012). Recombinant factor VIIa for the prevention and treatment of bleeding in patients without haemophilia. Cochrane Database Syst. Rev..

[28] Levi M., Levy J.H., Andersen H.F., Truloff D. (2010). Safety of recombinant activated factor VII in randomized clinical trials. N. Engl. J. Med..

[29] Abdul Sultan A., Grainge M.J., West J., Fleming K.M., Nelson-Piercy C., Tata L.J. (2014). Impact of risk factors on the timing of first postpartum venous thromboembolism: A population-based cohort study from England. Blood.

[30] Hart C., Bauersachs R., Scholz U., Zotz R., Bergmann F., Rott H., Linnemann B. (2020). Prevention of venous thromboembolism during pregnancy and the puerperium with a special focus on women with hereditary thrombophilia or pior VTE-position paper of the working group in women’s health of the Society of Thrombosis and Haemostasis (GTH). Hamostaseologie.

[31] Wikkelsø A.J., Edwards H.M., Afshari A., Stensballe J., Langhoff-Roos J., Albrechtsen C., Ekelund K., Hanke G., Secher E.L., Sharif H.F. (2015). Pre-emptive treatment with fibrinogen concentrate for postpartum haemorrhage: Randomized controlled trial. Br. J. Anaesth..

[32] Ducloy-Bouthors A.S., Mercier F.J., Grouin J.M., Bayoumeu F., Corouge J., Le Gouez A., Rackelboom T., Broisin F., Vial F., Luzi A. (2021). Early and systematic administration of fibrinogen concentrate in postpartum haemorrhage following vaginal delivery: The FIDEL randomised controlled trial. BJOG.

[33] Collins P.W., Cannings-John R., Bruynseels D., Mallaiah S., Dick J., Elton C., Weeks A.D., Sanders J., Aawar N., Townson J. (2017). Viscoelastometric-guided early fibrinogen concentrate replacement during postpartum haemorrhage: OBS2, a double-blind randomized controlled trial. Br. J. Anaesth..

[34] Pacheco L.D., Clifton R.G., Saade G.R., Weiner S.J., Parry S., Thorp J.M., Longo M., Salazar A., Dalton W., Tita A.T.N. (2023). Tranexamic acid to prevent obstetrical hemorrhage after cesarean delivery. N. Engl. J. Med..

[35] Tran H.T., Sørensen B., Rea C.J., Bjørnsen S., Ueland T., Pripp A.H., Tjønnfjord G.E., Holme P.A. (2014). Tranexamic acid as adjunct therapy to bypassing agents in haemophilia A patients with inhibitors. Haemophilia.

[36] Livnat T., Tamarin I., Mor Y., Winckler H., Horowitz Z., Korianski Y., Bar-Zakay B., Seligsohn U., Salomon O. (2009). Recombinant activated factor VII and tranexamic acid are haemostatically effective during major surgery in factor XI-deficient patients with inhibitor antibodies. Thromb. Haemost..

[37] Colucci G., Helsing K., Biasiutti F.D., Raio L., Schmid P., Tsakiris D.A., Eberle B., Surbek D., Lammle B., Alberio L. (2018). Standardized management protocol in severe postpartum hemorrhage: A single-center study. Clin. Appl. Thromb. Hemost..

[38] Woman Trial Collaborators (2017). Effect of early tranexamic acid administration on mortality, hysterectomy, and other morbidities in women with post-partum haemorrhage (WOMAN): An international, randomised, double-blind, placebo-controlled trial. Lancet.

[39] Surbek D., Blatný J., Wielgos M., Acs N., Edwards H., Erez O., Bartha J.L., Madar H., Mercier F.J., Schlembach D. (2024). Role of recombinant factor VIIa in the clinical management of severe postpartum hemorrhage: Consensus among European experts. J. Matern. Fetal Neonatal Med..

